# Toward Improved Diagnosis Accuracy and Treatment of Children, Adolescents, and Young Adults With Ependymoma: The International SIOP Ependymoma II Protocol

**DOI:** 10.3389/fneur.2022.887544

**Published:** 2022-06-02

**Authors:** Pierre Leblond, Maura Massimino, Martin English, Timothy A. Ritzmann, Lorenza Gandola, Gabriele Calaminus, Sophie Thomas, David Pérol, Julien Gautier, Richard G. Grundy, Didier Frappaz

**Affiliations:** ^1^Institut d'Hématologie et d'Oncologie Pédiatrique, Lyon, France; ^2^Fondazione IRCCS Istituto Nazionale dei Tumori, Milan, Italy; ^3^Birmingham Children's Hospital, Birmingham, United Kingdom; ^4^Children's Brain Tumour Research Centre, Medical School, Queen's Medical Centre, Nottingham, United Kingdom; ^5^Pediatric Radiotherapy Unit, Fondazione IRCCS Istituto Nazionale dei Tumori, Milan, Italy; ^6^Department of Pediatric Hematology and Oncology, University Hospital of Bonn, Bonn, Germany; ^7^Department of Paediatric Neuropsychology, Nottingham Children's Hospital, Queen's Medical Centre, Nottingham, United Kingdom; ^8^Department of Clinical Research and Innovation, Centre Léon Bérard, Lyon, France

**Keywords:** ependymoma, treatments, overall survival, progression free survival, randomized controlled trial

## Abstract

**Background:**

The clinical management of ependymoma in childhood and adolescence is complex and the clinicobiopathological correlates of outcome remain poorly understood. This international SIOP Ependymoma II (SIOP EPII) trial aims to improve the outcome of patients with ependymoma.

**Methods:**

SIOP EPII includes any patient <22 years at diagnosis with ependymoma, stratified by age, tumor location, and outcome of the initial surgery. Centralized pathology and imaging is required for diagnosis confirmation. SIOP EPII included three randomized studies according to age, postoperative residue, and suitability to receive radiotherapy. Patients ineligible for interventional strata are followed-up in an observational study. The staging phase aims to determine if central neurosurgical and radiological postoperative MRI reviews increase the resection rate. Patients ≥*12 months with* (i) *no residual disease* are randomly assigned in a phase III trial to evaluate the efficacy of post-radiation 16-week chemotherapy (VEC + CDDP) on PFS (stratum I); (ii) *centrally confirmed measurable inoperable residual disease* are allocated to randomized frontline chemotherapy phase II study (VEC vs. VEC + high-dose methotrexate) and considered for a second-look surgery (stratum II). If second-look surgery is not feasible or tumor residuum remains, patients receive 8 Gy-boost radiotherapy after conformal radiotherapy (phase I). (iii) Patients < *12 months (18 months in the UK) or not eligible to receive radiotherapy* are randomized in a phase II study to receive chemotherapy (alternated myelosuppressive and nonmyelosuppressive chemotherapy), with or without valproate (stratum III). To overcome the limitations encountered in the preliminary conclusions of the ACNS-0831 study, a SIOP EPII dedicated on-study amendment has been planned to definitively conclude the relevance of maintenance chemotherapy in stratum I. Secondary outcomes include overall survival, quality of life, neuropsychological and neuroendocrine outcomes, safety, and identification of key prognostic biomarkers (BIOMECA).

**Clinical Trial Registration:**

ClinicalTrials.gov, identifier: NCT02265770.

## Introduction

Pediatric ependymomas are enigmatic tumors that continue to present a clinical management challenge despite advances in neurosurgery, neuroimaging techniques, and radiation therapy. The predilection for very young children presents distinct management challenges and effective treatment of ependymoma remains one of the more difficult tasks in pediatric oncology. These malignant tumors arise throughout the CNS, but in childhood are located intracranially in 90% of cases, most frequently in the posterior fossa. Clinical management requires experienced multidisciplinary teams to propose the most appropriate treatment based on a thorough understanding of the biological diversity and prognostic implications. Surgical resection and the age at presentation are considered the most consistent prognostic factor for children with ependymoma. Serial studies have confirmed the importance of gross total resection (GTR) and focal radiotherapy in patients with localized disease resulting in 7-year overall survival rates of 75% and up to 85% in specific subgroups (1–6). Magnetic resonance imaging (MRI) scan should be performed within 24–48 h postsurgery to reliably evaluate the postsurgical status. GTR rates after initial surgery were around 50% in older studies such as the first SIOP Ependymoma study (7, 8). In patients with no residual tumor, the 5-year PFS is 50%−80% and drops to 0%−26% in patients with incompletely resected tumors (9, 10). Despite publications suggesting that a more intensive surgical approach improves GTR rates, there is no consensus on the optimal way to perform second-look surgery before radiotherapy and/or chemotherapy (3–5, 11, 12). The neurological consequences of an aggressive surgery should be carefully addressed (13), and surgical expert advice should be requested before undertaking second-look surgery if needed. The biology of these tumors is the subject of recent discoveries that may, in the future, become major prognostic and stratification factors for future trials and offer innovative therapeutic options (14, 15). This highlights the need for integrated biological studies. Despite optimal treatment, high rates of relapses are observed with ensuing significant risks related to tumor and treatment, and the prognosis of relapsed ependymoma remains poor (16). This approach aims at achieving and maintaining first complete remission, at the lowest possible cost to quality of life (QoL).

The international clinical program SIOP Europe Ependymoma II (SIOP EPII) was initiated in 2015 and aims to improve the accuracy of the primary diagnosis of ependymoma and define the most appropriate therapeutic strategies in children, adolescents, and young adults with ependymoma (NCT02265770). The program started shortly after the multicentric Children's Oncology Group (COG) NIH-supported randomized phase III trial ACNS 0831 (ClinicalTrials.gov Identifier: NCT01096368) which also examined the role of chemotherapy compared to observation following radiation therapy in young patients older than 1 year with newly diagnosed completely resected ependymoma. The treatment combining radiotherapy and chemotherapy has been recently reported in children older than 1 year following complete resection in ACNS 0831 trial, with benefits reported according to per-protocol analysis (17). While offering a relatively similar approach for patients over 1 year of age, the SIOP EPII program also provides novel treatment strategies for patients with residual disease (as defined in Table 1) despite chemotherapy and optimal safe surgery by investigating the benefit of a phase I radiotherapy boost of 8 Gy to tumor residuum alongside conformal radiotherapy dose of 59.4 Gy. It also offers an interventional study for an important group of very young children based on primary chemotherapy with the aim of avoiding or at least postponing the use of radiotherapy. More intensive surgery and upfront radiotherapy for children with aggressive ependymomas have improved survival outcomes, but this comes with a cost of 1.33 intellectual quotient (IQ) points lost per year (18). Indeed, the role of primary chemotherapy strategies is to preserve the immature brain of these children and protect cognitive functions that warrant further exploration, and the “Baby Brain” protocol was used in the UK from 1992 to 2004 without compromising overall survival or quality of life (19). Very young children with ependymoma initially treated with postoperative chemotherapy and then radiotherapy after relapse suffered more significant deficits in perceptual reasoning, full-scale IQ, word reading, and numerical operations than those who were spared radiotherapy (19). However, the potential impact of surgery on relapse was not clearly specified in this series (20).

**Table 1:** Refined SIOP EPII definitions of residual tumors (specific criteria that apply only for early postoperative MRI).

**R0**	**No residual tumor on postoperative MRI in accordance with the neurosurgical report**.
**R1**	**No residual tumor on MRI but description of a small residual tumor by the neurosurgeon or if the neurosurgical result is unknown**.
**R2**	**Small residual tumor on MRI with the maximum diameter below 5 mm in any direction**.
**R3**	**Residual tumor that can be measured in 3 planes**.
**R4**	**Size of the residual tumor not differing from the preoperative status (e.g., after biopsy)**.
**RX**	**If imaging is inadequate or the surgical cavity is very confusing, the term “unclear” should be possible. Every effort should be attempted to clarify the conclusion. Sometimes the presence of blood can be ruled out and distinguished from tumor if the MRI is repeated after some days. Repetition of MRI also may help to distinguish operative changes from residual tumor on T2-weighted-Fluid-Attenuated Inversion Recovery (T2/FLAIR)**.

## SIOP Ependymoma II Program

**Figure 1:**
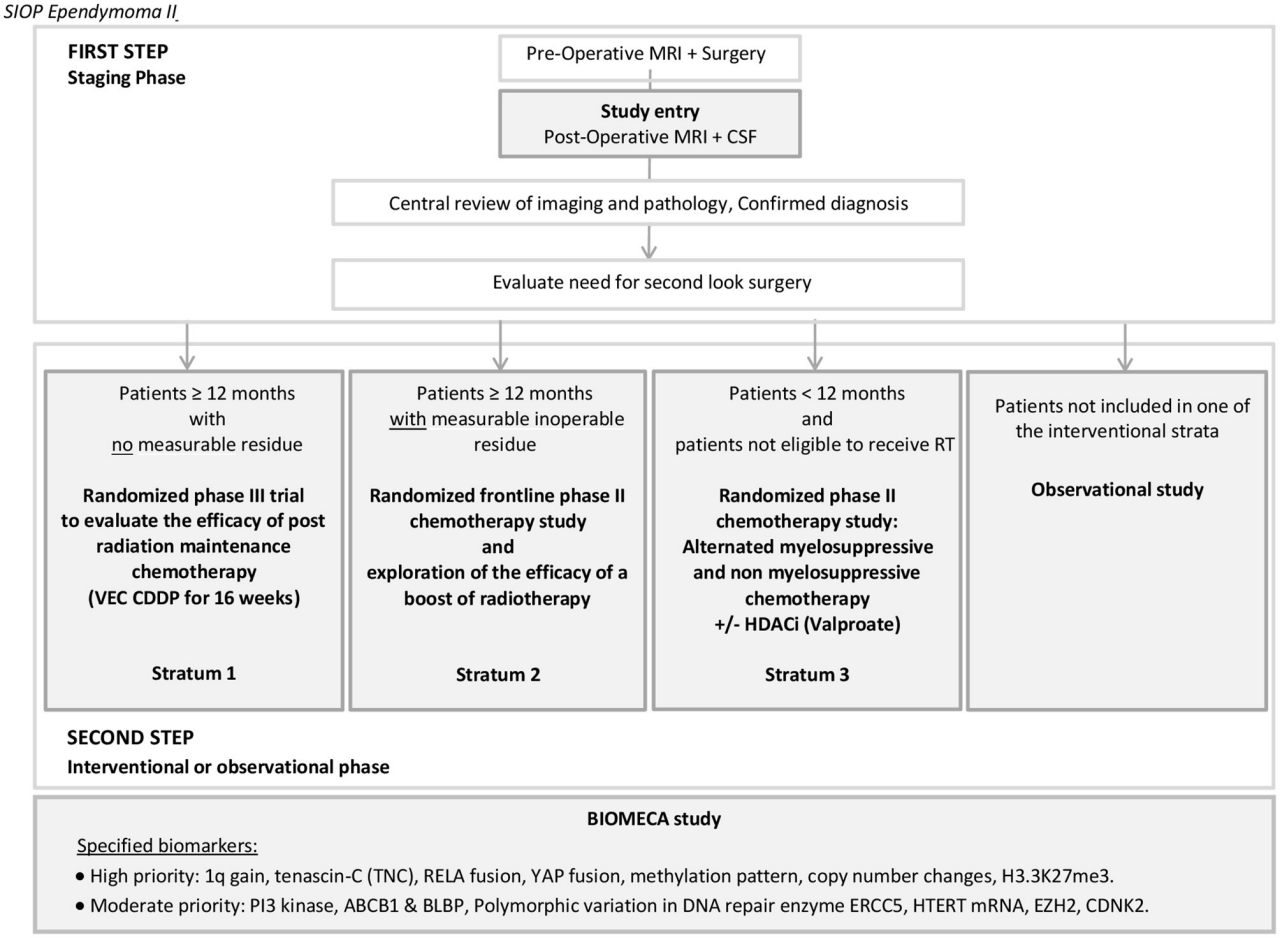
General scheme of the SIOP EPII trial. BIOMECA: Biomarkers of Ependymomas in Children and Adolescents.

**Figure 2:**
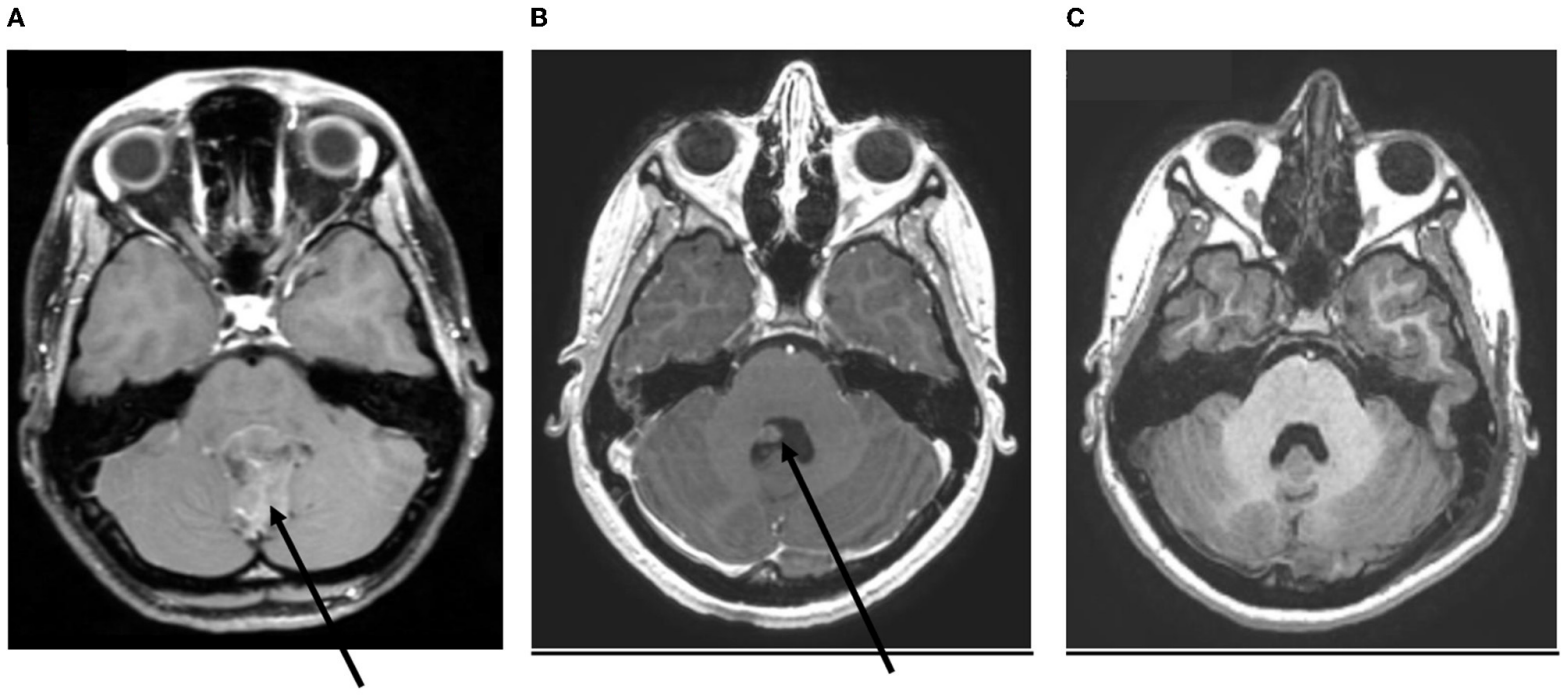
Imaging of one patient with ependymoma, at different times. **(A)** At diagnosis. **(B)** Immediate postoperative imaging. **(C)** Postoperative imaging after second-look surgery.

This position article presents the study protocol of the European SIOP Ependymoma II program (SIOP EPII) (Figure 1). All patients with ependymoma are eligible to be enrolled in the program after tumor documentation (biopsy or resection) and a centrally-confirmed pathology diagnosis of ependymoma. Patients are included in one of the three randomized interventional strata according to histological findings, metastatic status, age, surgical outcome, and eligibility for radiotherapy, or, alternatively can be enrolled in an observational study (Figures 1, 2).

**Figure 3:**
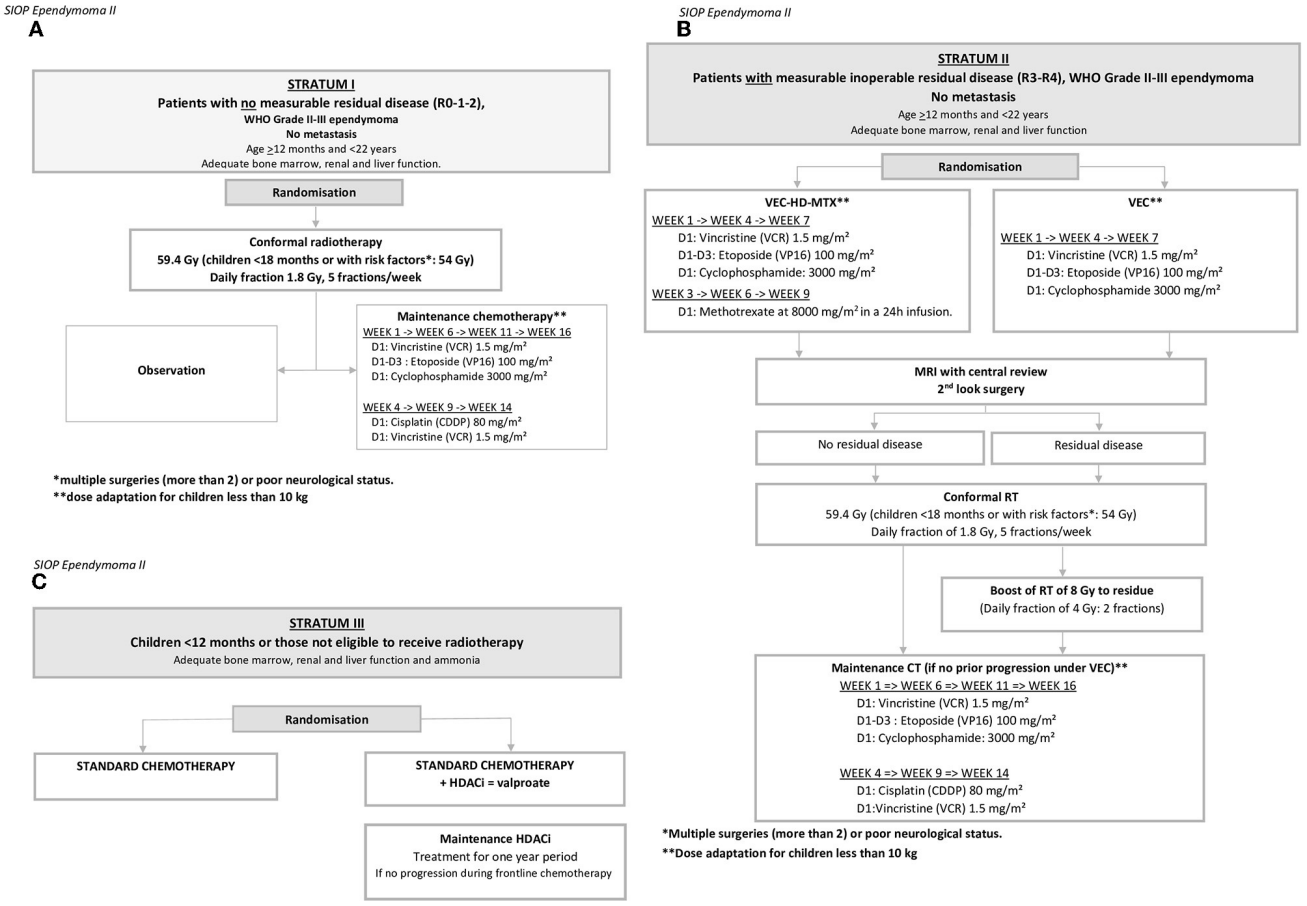
Scheme of the three interventional strata. **(A)** Stratum I: Patients with no measurable residual disease (R0-1-2). **(B)** Stratum II: Patients with measurable inoperable residual disease (R3–R4), WHO Grade II–III ependymoma, no metastasis. **(C)** Children <12 months or those not eligible to receive radiotherapy. *Multiple surgeries (more than 2) or poor neurological status; **Dose adaptation for children less than 10 kg. ^*^multiple surgeries (more than 2) or poor neurological status; ^**^dose adaptation for children less than 10 kg.

This ongoing trial seeks to test key hypotheses all aimed at improving outcomes:

1. There will be an improvement in patients who receive chemotherapy following complete surgical resection and radiotherapy compared to those that undergo complete surgical resection and radiotherapy alone (stratum I; Figure 3A, Table 2).
2. The addition of high dose methotrexate to the standard of care chemotherapy (VEC) improves progression-free and overall survival in patients with tumor residuum on imaging after surgery (randomized phase II) and this stratum will evaluate the role of “second-look” surgery in improving outcomes (stratum II) and determine the toxicity, feasibility, and efficacy of an 8 Gy boost in patients with residual disease (stratum II) (Figure 3B, Table 2) [a phase I study].
3. Adding valproic acid (VPA) as a histone deacetylase inhibitor to the primary chemotherapy strategy improves progression-free and overall survival in a randomized phase II study (stratum III; Figure 3C, Table 2).
4. Conformal radiotherapy does not adversely affect neurocognitive outcomes in very young children with ependymoma—secondary endpoint.
5. Key molecular events in pediatric ependymoma pathogenesis are predictive of clinical behavior and will allow future treatment stratification (precision medicine)—exploratory endpoint.

**Table 2:** Synthetic presentation of objectives and endpoints of the study for the staging, and different strata.

**STAGING**		
**Objectives**		
The primary objective is to determine whether centralized review of postoperative MRI can improve assessment of residual disease, increase the rate of complete resection compared to historical controls, and whether central neurosurgical and radiological review increase resection rates. Secondary objectives aim to evaluate second-look surgery rates as compared to historical controls.		
**Endpoint of the overall program**		
Primary endpoint is Gross Total Resection (GTR) rate.		
Secondary endpoint is second-look surgery rate. Only descriptive statistics are produced for GTR rate and second-look surgery rate.		
**Stratum I**	**Stratum II**	**Stratum III**
**Objectives**	**Objectives**	**Objectives**
The primary objective of the stratum I is to investigate whether PFS is improved in patients receiving 16-week chemotherapy (VEC + CDDP) after surgical resection and cRT compared to patients treated with surgical resection and cRT exclusively. Secondary objectives include assessment of overall survival (OS), neuroendocrine morbidity in each treatment arm, neuropsychological morbidity, quality of survival (QoS), and evaluation of safety in each treatment arm.	The primary objective is to compare the efficacy of the 2 postoperative chemotherapy schedules, VEC compared to VEC + HD-MTX in patients with incompletely resected ependymoma. Secondary objectives include safety and tolerability, evaluation of improvement in OS and PFS in patients who receive VEC + HD-MTX following surgical resection compared to those who receive VEC alone; to compare the neuroendocrine morbidity; to evaluate the neuropsychological morbidity; quality of survival in each treatment arm; to determine safety of 8 Gy boost radiotherapy in patients with residual disease after frontline chemotherapy and 59.4 Gy cRT.	For patients unable to receive radiation therapy, the primary objective is to evaluate the efficacy of adding the histone deacetylase inhibitor valproate to the standard chemotherapy regimen when compared to those that undergo chemotherapy alone. Secondary objectives include evaluation of OS and radiotherapy-free survival in patients in both groups; to evaluate neuroendocrine morbidity, neuropsychological morbidity, and QoS in each treatment arm; to determine the safety and tolerability of valproate combined to the standard chemotherapy in children not eligible to radiation therapy.
**Endpoints**	**Endpoints**	**Endpoints**
The primary endpoint is PFS calculated as the time from randomization to the date of event defined as progression or death due to any cause. Secondary endpoints included OS defined as the time from randomization to the date of death due to any cause, QoS, neuropsychological and neuroendocrine outcomes (late effects), short- and long-term safety. Efficacy is assessed using brain MRI performed within 6 weeks after the end of the radiotherapy for all patients, after cycle 2 and cycle 4 for patients with maintenance chemotherapy, then 3 months after the last radiological assessment for all patients. Ototoxicity must be evaluated at day 0 and before the 3^rd^ cycle of CDDP. Tolerance is assessed continuously for all patients. Adverse events are assessed and graded according to the Common Terminology Criteria Adverse Events (CTCAE v4.03).	The primary endpoint is the number of treatment responders. Objective response to chemotherapy is measured based on SIOPE Neuro Imaging guidelines. Secondary endpoints include OS calculated as the time from randomization to the date of death due to any cause, PFS calculated as the time from randomization to the date of event defined as progression or death due to any cause, QoS, neuropsychological outcomes, neuroendocrine outcomes (Neuroendocrine late effects), short- and long-term safety Adverse events are assessed and graded according to the Common Terminology Criteria Adverse Events (CTCAE v4.03). Exploratory endpoints include toxicity monitoring in the subgroup receiving radiotherapy boost, and event-free survival (EFS) in patients receiving a radiotherapy boost.	The primary endpoint is PFS calculated as the time from randomization to the date of event defined as progression or death due to any cause. Secondary endpoints included OS defined from randomization to the date of death due to any cause; Radiotherapy-free survival rate; QoS; Neuropsychological outcomes; Neuroendocrine outcomes (late effects); Short- and long-term safety. Adverse events are assessed and graded according to the Common Terminology Criteria Adverse Events (CTCAE v4.03).

The interventional stratum I (Figure 3A, Table 2) aims to evaluate the clinical impact of 16-week chemotherapy (vincristine, etoposide, and cyclophosphamide (VEC) + Cisplatin (CDDP) following surgical resection and conformal focal radiotherapy (cRT) in patients older than 1 year *with centrally confirmed complete resection* of intracranial ependymoma. The proposed chemotherapy schedule uses the combination of vincristine-etoposide-cyclophosphamide (VEC) and cisplatin (CDDP), drugs currently providing the best response rate at the time of protocol design (21).

Stratum II (Figure 3B, Table 2) enrolls patients older than 1 year, *with measurable residual disease*, that are centrally confirmed to have residual disease, despite optimal safe surgery and investigates the efficacy of VEC chemotherapy combined with high-dose methotrexate (HD-MTX) vs. VEC chemotherapy. All pre- and postoperative MRIs are reviewed and discussed in national or international committees including radiologists and neurosurgeons to gain consensus on the feasibility/morbidity of second-line surgery. Based on MRI response assessment to chemotherapy, further surgery is again discussed at the national level. All patients receive cRT. Patients with the persistent residual inoperable disease after induction chemotherapy receive an additional 8-Gy boost of radiotherapy to the residual tumor, delivered immediately after cRT. The aim of the phase I study of an 8 Gy boost of radiotherapy is to optimize local control, extend overall survival while preserving patient quality of life, and evaluate safety in a multicenter setting (21).

Stratum III aims to evaluate the benefit of postoperative chemotherapy administered alone or combined with the histone deacetylase inhibitor (HDACi) valproate in very young children (<12 months) or in patients not eligible for radiotherapy (Figure 3C, Table 2).

Patients who are not eligible for one of the three randomized interventional trials, or children whose parents are unable to give informed consent to the interventional study may be included in the observational study to increase global understanding of the disease. Furthermore, the optimal treatment and management of children with spinal ependymoma is still unclear and active recruitment in the observational arm of the umbrella study attempts to address this. It is particularly important for future trials to be able to propose the optimal treatment for these patients with frequently difficult to manage tumors. An improved understanding of this disease will allow us to better define the most appropriate treatments, and help to refine future trial designs. Indeed, the recognition that myxopapillary tumors are less indolent than hitherto thought, has led to WHO proposing these are in fact grade II tumors, underlines the importance of including these patients within the umbrella of a clinical trial (22). In the same way, including patients with metastatic and/or relapsing disease in the staging phase of the program then in the observational study could provide substantial information on the tumor biology and treatment of specific high-risk patients with very poor prognosis (16, 23).

The necessity of international expertise is becoming increasingly obvious, and improved access to international experts is highly encouraged; referrals could be facilitated through the constitution of the European reference network (ERN) and more specifically in pediatrics through existing networks such as the ERN PaedCan (https://paedcan.ern-net.eu/home/about-ern-paedcan/). EURACAN members recently reported progressive integration to health care systems of member states in the ERN for adult solid cancers (24). Such networks may also help in the pathological review of these patients, taking into account that results from the SIOP EPII database reported 3.3% of misdiagnosed patients.

This large comprehensive international program with a potentially more intensive surgical strategy assesses the risk of neurological morbidity. Furthermore, the adoption of a lower age of radiation (from 36 months) brought with it a duty of care to evaluate the impact of radiotherapy and neuro-cognitive development of very young children who had, in different countries, hitherto received primary chemotherapy to avoid or delay irradiation (11, 20, 25). This will, in turn, allow a nuanced view of the relative benefit of different treatment strategies in this very young age group. In response to this and to accommodate the differing levels of neuropsychology input across European partners, the SIOP EPII development and implementation of the cognitive test protocol Core-Plus model for international brain tumor trial has recently been published. Neurocognitive development and other aspects of quality of survival are captured through the SIOPE Core Plus model (26). This comprehensive battery of cognitive tests and quality of survival measures is implemented at postsurgical baseline, 2- and 5-year follow-up, and at age 18 years, as recently reported (27). The Core Plus model circumvents resource discrepancies within and between participating countries by mandating a minimum Core battery of measures augmented by more comprehensive measures where feasible.

Previous studies reported rates of patients with PF-EPN-A, PF-EPN-B, and ST-EPN-RELA (or ZFTA)-fused, and ST-EPN-YAP1-fused subtypes of approximately 80, 20, and 80%, and <10%, in each compartment, respectively (4, 14, 21, 28). The integrated biological study *Biomarkers of Ependymomas in Children and Adolescents* (BIOMECA) is dedicated to testing and validating the hypothesis of the predictive value of biomarkers in specific subgroups of ependymoma on their clinical and biological behaviors. This study will prospectively evaluate 1q copy-number status, Tenascin C, RELA (or ZFTA)-fusion, YAP1 fusion, H3.3K27me3, and molecular subgroups (methylation array) as prognostic and predictive biomarkers in ependymoma within the clinical trial (Figure 1). Moreover, the BIOMECA study proposes to explore and identify new biomarkers for ependymoma clinical behaviors (location, recurrence, chemoresistance, and metastasis). Despite no specific statistical design being initially prespecified (study conception was performed before 2014), the dedicated on-study amendment stipulated that the study will explore survival in the different molecular subtypes to conclude which molecular subgroups would benefit from maintenance chemotherapy (stratum I). The first preliminary results are expected in the coming months.

## Current Study Status

The current protocol used version 3.1 dated, 22nd April 2020, as presented in Supplementary Material S1, Supplementary Tables S2, S3).

The SIOP EPII program opened in 2015 in France and the UK, then Italy and Belgium in 2016, Spain, Ireland, and the Czech Republic in 2017, Austria, Switzerland, Finland, and Germany in 2018, the Netherland, Denmark, and Norway in 2020, and remaining participating countries continue to open (Supplementary Table S4).

As of 31st August 2021, 160 investigational sites have been activated in over 14 participating countries. Since 23rd June 2015, 580 patients have been included in the whole program (Supplementary Figure S5). The second substantial amendment displayed the staging phase is mandatory for all newly diagnosed patients. Some patients entered the observational stratum before the amendment was applied (without staging, *N* = 21), or with staging (*N* = 187). A total of 46 patients were excluded from the program during the staging phase (mainly ependymoma diagnosis not confirmed by central pathological review, consent withdrawal, investigator decision, disease progression during staging, noncompliance) and 306 patients have been allocated for randomization in one of the three interventional strata out of the 480 patients expected (stratum I *N* = 224, stratum II *N* = 35, and stratum III *N* = 47; Supplementary Figure S5).

## Discussion

This large European program SIOP EPII aims to prospectively enroll all patients with centrally confirmed histological ependymoma after resection and proposes an integrated strategy of treatment and knowledge acquisition. This umbrella program allocates patients according to histological findings, metastatic status, age, postsurgery outcome, and eligibility for radiotherapy to one of the three proposed randomized studies to evaluate different therapeutic strategies, or to an observational arm.

Building on previous results from retrospective studies, and evidence from scarce randomized studies, the SIOP EPII international program relies on a network of European experts in the field of ependymoma. Trials at the national level are frequently limited by the scarcity of patients and are not adequate in the context of rare childhood cancers; consequently, large-scale international trials are required. Based on the international collaboration, refined trial designs and higher levels of proof can be achieved. Key opinion leaders were commissioned by the SIOP as SIOP EPII chief investigators and national coordinators, who strongly supported the development of the umbrella program, worked together to find a consensus, and defined the most appropriate therapeutic strategies. A strong consensus emerged on the need to improve diagnosis accuracy at the primary clinical presentation in patients with ependymoma. Support from a central review bringing together multidisciplinary European experts may be helpful to provide the most appropriate care to all patients.

Such an international program guarantees the most reliable diagnosis regardless of the initial point of entry, and access to experimental treatment is considered by experts as the most appropriate. While patients benefit from treatment, their participation also contributes to treatment evaluation and to the most up-to-date experience provided through multidisciplinary international organizations.

The strength of the SIOP EPII program is to provide central pathology and central imaging reviews for any patient presenting with newly diagnosed ependymoma, gathering experts in each specific field, alongside a comprehensive treatment strategy for the myriad presentations and clinical complexity of ependymoma in childhood and adolescence. The three randomized studies are currently recruiting and the recruitment rates are now consistent with the theoretical expectations notwithstanding the regulatory constraints some European countries faced to get investigational sites opening authorization, and patients from 14 out of the 18 identified countries are currently eligible for recruitment. Suspension of inclusions related to the COVID-19 pandemic for 1–4 months occurred in some countries particularly Germany, Austria, and France and no significant impact on accrual rates has been reported.

One of the constraints related to the long-term development of international programs is that emergent results and advances in knowledge gathered over years may jeopardize or challenge the long-term viability of expected results. Conversely, it can also highlight the importance of continuing and completing such studies, with highly expected results to definitively address the role of chemotherapy in completely resected ependymoma. The preliminary results from the COG phase III trial ACNS-0831 studying chemotherapy compared to observation following radiation therapy in young patients (>1 year) with a completely resected ependymoma recently suggested a potential clinical benefit in favor of adjuvant post-radiation chemotherapy and showed significantly improved event-free survival (17). While the results of ACNS-0831 intention-to-treat analyses showed a trend in favor of treatment combining radiotherapy and chemotherapy in these patients, preliminary results of “per-protocol” analyses confirmed a significant clinical benefit in favor of treatment combining radiotherapy and chemotherapy for children older than 1 year following complete resection. Unfortunately, marked noncompliance to the trial has been reported with 30% of patients allocated to randomization to chemotherapy arm who finally did not receive chemotherapy. Results should be appropriately confirmed to avoid premature prejudice regarding the use or avoidance of chemotherapy in completely resected ependymoma. To overcome similar issues, we planned a dedicated on-study amendment to ensure that the study will be correctly powered based on the intent-to-treat population, and will allow us to draw conclusions on the relevance of maintenance chemotherapy in patients included in the stratum I. Only results from further large trials would allow addressing the appropriate treatment in these patients with ependymoma, and, namely, determine to what extent chemotherapy provides a survival advantage. The treatment strategy in SIOP EPII stratum I is highly similar, and results will provide reliable final results, and constitute an unparallel dataset, and a combined cohort with COG to explore treatment outcomes and biology in approximately 640 children with completely resected ependymoma. Furthermore, the other strata and observational studies in the SIOP EPII umbrella will provide innovative and unique results.

Recruitment to stratum I in SIOP EPII has been dampened by a relatively low randomization rate of 70%, based on the SIOP EPII noninterventional study results. As of August 2021, the observation group showed that 94 out of the 114 patients older than 12 months, R0, R1, and R2 (Table 1) were potentially eligible in stratum I, even though we cannot exclude that the other inclusion criteria were not fulfilled. Such rates translate difficulties to collect informed consent for an experimental strategy implying longer and more complex treatment along with additional toxicities and patient risks. For a child eligible for stratum I, families received advice from the primary clinician and bias regarding the randomization is likely to be introduced. Indeed, bias regarding chemotherapy may color this discussion. The recent per-protocol results of the COG study provided evidence for the potential benefit of chemotherapy in completely resected ependymoma, it is hoped that they will further support trial entrance and compliance to address this important issue. The preliminary evidence for a potential benefit of chemotherapy reported in the COG trial may support increased randomization of patients with ependymoma to stratum *I* (17). We recently noted an increased number of randomized patients in the past few months in 2021 with nearly six patients/month randomized since January 2021 compared to 3.75 patients/month in 2020.

There can, however, be little doubt that the prognosis for relapsed ependymoma is dismal with an approximately 25% cure rate (8, 16). The chance to improve outcomes must be grasped at the first presentation which, in turn, has to be through recruitment to interventional studies seeking to achieve this end. Furthermore, the “observation group” aims to include patients with spinal ependymoma, metastatic disease, and relapses and will contribute to improving knowledge in clinical outcomes and tumor biology and provide innovative data for future trials.

Patients with incompletely resected ependymoma have a poor prognosis, and stratum II investigated the randomized comparison of standard of care vincristine, etoposide, and cyclophosphamide (VEC) vs. VEC plus high-dose methotrexate. The study also tries to identify the role of second-look surgery after chemotherapy at a national level. As suggested by the UK organization Ependymoma Multidisciplinary Advisory Group EMAG, collaborations that facilitate the sharing of expertise and improve expert identification should be encouraged at the European level, possibly supported by the current development of networks such as ERN PaedCan. These international collaborations may contribute to improve access to optimal diagnosis through centralized pathological and imaging reviews, implementing quality control for radiotherapy, and providing evidence-based treatment guidelines to improve outcomes for young patients with ependymoma. For children in whom complete resection is not achieved at this point following centralized radiological review, an experimental 8 Gy-boost in addition to 59.4 Gy of conformal RT is being evaluated, such as Italian centers data suggested a survival advantage after RT boost (5). Results from a larger international study are expected to confirm the benefit of an 8 Gy-boost.

Ependymoma has a predilection to arise in very young children, indeed half of all cases occur under 5 years of age. A treatment stratum dedicated to this rare but challenging group of children was critical. Based on a meta-analysis of the outcomes of European Baby-brain strategies (UK, France, Italy, and Germany (D Malkin personal communication), the UK baby brain strategy provided superior outcomes and was adopted as the standard of care. In the absence of novel therapeutic strategies based on underlying biology, an epigenetic modifier was sought. Limited availability of “oven-ready” possibilities led to the adoption of the histone deacetylase inhibitor sodium valproate and a randomized study was proposed. The philosophy of using a primary chemotherapy strategy to ideally avoid or at least delay radiotherapy is somewhat controversial, with different European countries adopting different age ranges for inclusion in stratum I. The UK has remained concerned about the limited information on the neurocognitive damage of conformal radiotherapy to the posterior fossa and supratentorial area advocates entry at 18 months of age vs. 12 months in many other countries and over 36 months for supratentorial disease. Consequently, for very young children with supratentorial ependymoma, the umbrella protocol allows the inclusion of patients considered ineligible for radiotherapy on the basis that even with proton beam radiotherapy the quality of survival, due to neurocognitive damage of irradiating the supratentorial component of the developing brain (19), may be poor. Parental involvement in these difficult discussions is key as they, along with the child for whom they advocate, experience the real-life consequences of brain tumor therapy on a daily basis. A nuanced and informed discussion over the cure and importantly the cost of the cure is absolutely essential for very young children with ependymoma.

Patients with metastatic and/or relapsing disease included in the staging phase of the program then in the observational study have a worse prognosis (6, 23) and would benefit from the results of the stratum I and stratum II along with BIOMECA analyses. These results combined with other large molecular profiling will help in the design of new dedicated personalized studies. In parallel, the study MEMMAT (NCT01356290) is exploring an innovative antiangiogenic and intraventricular chemotherapy strategy in patients with recurrent ependymoma and opens new management opportunities for metastatic patients.

## Data Availability Statement

The original contributions presented in the study are included in the article/Supplementary Material, further inquiries can be directed to the corresponding author.

## Ethics Statement

The studies involving human participants were reviewed and approved by European Voluntary Harmonized Procedure (VHP358-VHP201385). Written informed consent to participate in this study was provided by the participants' legal guardian/next of kin.

## Author Contributions

All authors listed have made a substantial, direct, and intellectual contribution to the work and approved it for publication.

## Funding

This work was supported by the French Health ministry (PHRC-2012 and PHRC-2019), Societé Française des Cancers de l'Enfant, Enfant et Santé, and patients' association. Within the UK, this trial is funded by CRUK, and RG and TR are funded by Fighting ependymoma, Joe Foote Foundation, and Brains Trust UK. In Italy, grants were received from Associazione Bianca Garavaglia Onlus, Busto Arsizio (VA), Associazione Italiana per la Ricerca sul Cancro (AIRC) and Associazione Bimbo Tu, Bologna, Italy. The trial was supported at the national level by national grants or patients' associations.

## Conflict of Interest

The authors declare that the research was conducted in the absence of any commercial or financial relationships that could be construed as a potential conflict of interest.

## Publisher's Note

All claims expressed in this article are solely those of the authors and do not necessarily represent those of their affiliated organizations, or those of the publisher, the editors and the reviewers. Any product that may be evaluated in this article, or claim that may be made by its manufacturer, is not guaranteed or endorsed by the publisher.
