# Supplementary material for: Toward Improved Diagnosis Accuracy and Treatment of Children, Adolescents, and Young Adults With Ependymoma: The International SIOP Ependymoma II Protocol
Source: Front Neurol. 2022 Jun 2;13:887544. doi: 10.3389/fneur.2022.887544 (PMC9201444; doi:10.3389/fneur.2022.887544)
Supplement: Supplementary file 1 [file Table_1.DOCX]

**Supplementary materials S1. Participants, overall study design, methods for the staging phase and all interventional strata, statistics and sample size, follow-up, ancillary and transversal studies (all strata)**

**Participants**

Eligible patients were children, adolescents and young adults below the age of 22 years, diagnosed with ependymoma, attending one of the authorised centres in European participating countries.

All patients with ependymoma are enrolled in a staging phase before subsequent enrolment in one of the three interventional strata or in the observational study (Figure 1).

After tumour surgery, patients in staging phase undergo central review of imaging and pathology. Patients are then included in one of the three subsequent interventional strata or in the observational study, according to the outcome of initial resection (residual disease versus no residual disease), age, and suitability to receive radiotherapy. The three interventional therapeutic strategies proposed according to patient status are.

Stratum I (randomised phase III): patients aged 12 months or older, with no measurable residual disease

Stratum II (randomised phase II): patients aged 12 months or older, with measurable residual disease

Stratum III (randomised phase II): patient younger than 12 months or ineligible to radiotherapy

Observational stratum: patients who do not enter one of the interventional strata

Inclusion and exclusion criteria for the staging phase and each stratum are presented in Supplementary Table S2.

Patients and/or legal representatives who consent to participate must provide a written informed consent for the staging phase and a second written consent specific for further enrolment into interventional studies.

**Overall study design**

The comprehensive international program SIOP EPII (NCT02265770) was approved by the International Society of Paediatric Oncology (SIOP) Europe and displays SIOP Europe label. The Centre Léon Bérard, as sponsor, leads the global European coordination of the trial. The SIOP EPII program is performed according to the declaration of Helsinki and the International conference on Harmonisation on Good Clinical Practices. The study planned to recruit patients from 18 European countries. The multinational SIOP EPII clinical trial followed the European Voluntary Harmonised Procedure (VHP) VHP358-201385), ensuring a centralised assessment of the benefit/risk ratio of the clinical trial at the European level for all the participating member states through a single procedure. The VHP helps to address the issues that arose from implementation of the EU Clinical Trials Directive 2001/20/European Commission in 2004 into national laws and regulations, and to overcome divergent review processes for clinical trial applications in the EU member states. This harmonised European-wide approach for the approval of clinical trial applications has been chosen in the context of multinational clinical trials involving various National Competent Authorities and ethics committees. The VHP aims to achieve faster approvals through accelerated assessment of multinational clinical trials applications and to speed up global implementation of multinational clinical trials. After approval from a single European reviewer (randomly allocated), each national coordinating centers proceed with national competent authorities and ethics committees to get final approval according to national laws.

Each stratum is conducted by a dedicated chief investigator. Each national coordinator/national coordinating centre appointed specific national experts or national reference centre to coordinate dedicated issues: radiotherapy, biopathology, neuroradiology, neurosurgery, neuropsychology and Quality of Life (QoL). National coordinator/national coordinating centres maintain an overview of the study at each national level, and national coordinators participate in the steering group dedicated to oversee all the study. The sponsor delegated to national coordinators tasks and duties, and enter into mutual agreement for intellectual property, and publication rules.

Trial management

The trial uses specific national procedures and national experts for central reviews in place at trial initiation or requested for their specific implementation prior to national activation, in order to perform consistent assessment of patients’ characteristics over countries.

National coordinating centers (either academic clinical research teams or contract research organisations) are responsible for the coordination of the trial at each national level (sites selection and study initiation visit), and quality control of study data according to a predefined harmonised monitoring plan. National coordinating centers ensure that study is conducted according to the protocol requirements and recommendations for Good Clinical Practices of the International Conference of Harmonisation (ICH E6-R2).

The sponsor provided an electronic Case Report Form (Medsharing EOL software, Fontenay-Sous-Bois, France) to centralize data collection. Each national coordinator agreed for financial participation prorated for respective accrual and data management.

Independent Data Safety and Monitoring Committee (iDSMC)

An independent Data Safety and Monitoring Committee (iDSMC) was set up prior to trial initiation, and gathered three international independent experts: one methodologist/statistician and two medical experts, not otherwise involved in the SIOP EPII trial. The iDSMC has access to all relevant information in order to assess the benefit risk ratio of the study: accrual rate, safety data, results from interim analyses. A pre-specified number of events triggers IDSMC meeting, and guarantees project management. The iDSMC may recommend trial discontinuation if the recruitment rate or data quality are unacceptable or if any issues which may compromise patient safety are identified. If appropriate, iDSMC reports and study progress reports should be prepared every 6 months during the first 2 years, and then once a year in line with iDSMC approval.

**Staging phase**

The staging phase includes all patients and requires an exhaustive review of their medical history and a physical examination and neurological assessment (Behavioural Assessment and Research System test). Tumour samples (formalin-fixed/paraffin embedded block (FFPE)), lumbar puncture samples (Cerebrospinal fluid (CSF)) and Brain/spine MRI with gadolinium must be made available. Tumour samples and perioperative imaging (preoperative cranial and spinal MRI and postoperative cranial MRI) are centrally reviewed by national reference experts to confirm the pathological diagnosis and to determine the outcome of the initial surgical resection(s), in particular evidence of residual tumours after resection (Table 1). An inclusion in the biological study BIOMECA is also proposed, requiring tumour analysis, and an additional blood sample and CSF (taken at the time of the lumbar puncture for staging).

At the end of the staging phase, patients may be proposed to enter in one of the interventional strata (Figure 1). Of note, patients who are not eligible in one of the interventional strata may be included in the observational study. For patients with tumour residue (R3 or R4) evidenced radiologically (Table 1), and considered as non resectable by the local surgeon, the feasibility of a second look surgery is centrally assessed by one of the pre-identified experienced neurosurgeons, in charge of the second-look surgery. To illustrate the approach, a case report presenting imaging of one patient with newly diagnosed ependymoma is presented. (Figure 2).

Synthetic presentation of objectives and endpoints for the staging are presented in Table 2.

**Sample size**

A minimum of 480 patients will be enrolled in the overall program according to the statistical considerations presented below for each stratum.

**Interventional Stratum I- Patients with no measurable disease and ≥12 months of age (Phase III)** (Figure 1, Figure 3)

Patients aged ≥12 months and younger than 22 years with no residual measurable tumour (R0, R1, or R2) and no metastasis may enter the interventional stratum I. Eligible patients are allocated for randomisation to receive either conformal radiotherapy (cRT, standard strategy) or cRT followed by a 16-week maintenance chemotherapy (experimental strategy). Randomisation in stratum I is stratified on the tumour location (supra or infratentorial), grading (classic or anaplastic) and age (≤ or >3 years old at diagnosis). Objectives and endpoints for the stratum I are presented in Table 2.

**Study treatment**

Conformal Radiotherapy (cRT): within ≤6 weeks post-surgery, all patients receive, cRT of 59.4 Gy (only 54 Gy if <18 months and/or with risk factors such as multiple surgeries (more than 2) and/or poor neurological status) with a 0.5 cm margin in a 1.8 Gy daily fractions, 5 fractions per week (under general anaesthesia, if required). Patients, according to randomisation receive cRT followed by either:

16-week chemotherapy (VEC+CDDP) within 4 weeks after completion of standard 59.4 Gy radiotherapy, or surveillance (Supplementary Table S4).

A prospective quality control of radiotherapy has been implemented nationally to review treatment plans before irradiation initiation, and identify potential deviations to dose prescription, dose uniformity or volume. Specific dose modifications are recommended in the clinical trial protocol to prevent haematological, neurological, renal and audiology toxicities.

**Statistics, sample size.** This stratum is designed to detect an improvement of the 5-year PFS rate of 15% within the chemotherapy arm as compared to the observation arm (75% *versus* 60%; Hazard ratio=0.56). A total of 109 events would have 85% power to show statistically significant improvement in PFS at a 2-sided 5% alpha level. Assuming 10 years of recruitment, 3 years of follow-up for the last included patient (i.e. 13 years for total study duration), and two interim analyses for futility after 37 and 74 events, 160 patients per arm are required.

A total of 320 eligible patients will be randomised.

Randomisation 1:1 stratified by tumour location (supra versus infratentorial), grading (classic versus anaplastic) and age (≥ or <3 years old at diagnosis) into the two treatment arms (i.e. 160 patients per arm)

**Interventional stratum II– Patients with inoperable measurable residue and** ≥ **12 months of age (phase II)** (Figure 1 and Figure 3)

This study is designed as a phase II trial for patients with residual disease to investigate the possible activity of HD-MTX by giving to all patients the benefit of VEC chemotherapy whilst randomising half of patients to receive additional HD-MTX (Figure 3, Table S4). Then all patients receive cRT.

Patients with a residual inoperable disease after induction chemotherapy and cRT, are administrated an 8 Gy boost of radiotherapy to the residual tumour, immediately delivered after the end of the cRT to maintain local control and extend overall survival whilst maintaining the patient quality of life. Safety of boost radiotherapy will be evaluated.

Maintenance chemotherapy (Figure 3, Table S4) is given for patients not progressive during immediate post-operative chemotherapy.

Objectives and endpoints for the stratum II are presented in Table 2.

**Study treatment**

Postoperative induction chemotherapy: Patients receive an 8-week course of chemotherapy: VEC+HD-MTX or VEC. Chemotherapy should be initiated within 3 weeks after surgery.

Second look surgery: central review of the post induction chemotherapy MRI is performed for second look surgery whenever possible,

Conformal Radiotherapy (cRT): All patients receive cRT of 59.4 Gy (only 54 Gy for patients <18 months or with risk factors (more than 2 surgeries) or with poor neurological status) with a 0.5 cm margin in a 1.8 Gy daily fraction, 5 fractions per week (under general anaesthesia if required).

Boost of cRT (if residual tumour): patients with residual tumour despite pre-radiation chemotherapy and cRT will receive a boost of 8 Gy of cRT to the tumour residue (2 fractions of 4 Gy on 2 consecutive treatment days).

16-week maintenance chemotherapy: All patients whom disease did not progressed under first chemotherapy receive 16-week maintenance chemotherapy with VEC+CDDP.

A prospective quality control of radiotherapy has been implemented nationally to review treatment plans before irradiation initiation, and identify potential deviations to dose prescription, dose uniformity or volume. Specific dose modifications are recommended in the clinical trial protocol to prevent haematological, neurological, renal and audiology toxicities.

**Statistics, sample size.**

This stratum II has been designed as a single stage randomised phase II Jung design. Due to the rarity of the target population, the sample size is limited. It is anticipated that during the 10 years of recruitment, it will be possible to randomise at least 60 patients - 30 patients per arm. With 30 patients per arm, under the assumption that the true response rates are 30% in the control arm and 45% in the methotrexate arm then the suggested analysis rule below will lead to 79% power to successfully carry forward methotrexate for further evaluation, assuming a 34% risk that it will be carried forward when in fact it is ineffective. Annual interim analyses for safety and activity are planned using Pocok-type boundary.

A total of 60 eligible patients will be randomised

Randomisation 1:1 into the two treatment arms (VEC alone or VEC+HD-MTX)

Final analysis is planned 6 months after last patient randomisation

**Interventional stratum III– Randomised phase II chemotherapy study in children <12 months of age or those not eligible to receive radiotherapy** (Figure 1 and Figure 3, Table 2, and Supplementary Table S4)

This stratum is designed as a phase II trial to evaluate the benefit of postoperative dose intense chemotherapy administered alone or in combination with valproate. The aim is to minimise the risk of drug resistance whilst maximizing the intensity of treatment in very young children.

Objectives and endpoints for the stratum III are presented in Table 2.

**Study treatment**

Patients are randomised to receive chemotherapy combined or not with valproate. Chemotherapy should be initiated as soon as patient recovered from surgery and ideally within 3 weeks after surgery. The complete course of chemotherapy comprises 4 administrations of alternating myelosuppressive and relatively non-myelosuppressive drugs repeated every 56 days for a total of 7 cycles alone or in association with valproate.

At the end of chemotherapy, patients who have received valproate continue to receive valproate for an additional year.

**Statistics, sample size.** This stratum aims to recruit a minimum of 100 patients over 10 years, with 2.5-year follow-up at the end of the study. Median follow-up will therefore be 5 years.

Assuming that the true 5-year PFS rates are 45% and 60% in the control and valproate arms, respectively, then the study will have 80% power to detect the difference between the arms at a 1-tailed alpha level of 0.25. If recruitment is less than target, and only 82 patients are recruited, then the study retains 75% power to detect this difference. Conversely, if the study manages to recruit 130 patients, then it will have 80% power to detect the difference at a 1-tailed alpha level of 0.2.

A minimum of 100 eligible patients will be randomised

Randomisation 1:1 into the two treatment arms

**Follow-up after treatment (all strata)**

All patients from the interventional strata will be followed-up till 5 years after the completion of treatment to assess potential relapse of the disease (brain and/or spine MRI).

Neuroendocrine morbidity and QoS evaluations will be evaluated until age 18. Other investigations (namely those regarding ototoxicity) will be performed according to local practices and collected into database.

**Ancillary and transversal studies (all strata)**

The SIOP EPII clinical protocol includes the exploration of prognostic biomarkers. Key molecular events may help to predict clinical behaviour of ependymoma, prospectively evaluate 1q copy-number status, tenascin C, RELA-fusion, YAP-fusion, H3.3K27me3 and molecular subgroup (by methylation array) as prognostic and predictive biomarkers in ependymoma within clinical trial setting. Other objectives include identification of new biomarkers for Ependymoma clinical and biological behaviour (location, recurrence, chemo resistance, invasion, metastasis), validation of known biomarkers in this international prospective study for newly diagnosed ependymoma, definition of new comprehensive grading scheme based on the histological, and immunohistochemical and/or biological criteria that could be used for newly diagnosed ependymoma, validation and comparison of techniques to assess biomarkers for further use in the stratification of patients, and selection of the best biomarker(s) and definition of a prognostic signature for ependymoma.

This part of the program relates to the European ependymoma biology consortium “Biomarkers of Ependymomas in Children and Adolescents (BIOMECA)”, and reached a cooperation agreement which aims to identify informative prognostic biomarkers for assessment of disease status and predictive response to therapy. These results will be published separately.

neuropsychological, neuroendocrine morbidity, cognitive morbidity and QoS/QoL are evaluated in all the interventional strata. Development and initial implementation of a cognitive test protocol have been recently presented separately (Thomas *et al.*, 2019)

**Exploratory Endpoints**

Pharmacokinetic modelling will be carried out using valproate pharmacokinetic parameters in conjunction with patient characteristics and clinical parameters in order to investigate the key factors involved in determining individual valproate drug exposures within the patient population.

Valproate pharmacodynamics will be followed throughout, monitoring changes in histone H3 and H4 acetylation. Changes between baseline and time of steady state valproate will be correlated by trough levels and clinical response.

| **Supplementary Table S2. Inclusion and exclusion criteria.** | | | |
| --- | --- | --- | --- |
| **Staging phase** | | | |
| **Inclusion criteria**   - Main residence in one of the participating countries - Age <22 years old at the diagnosis - Histological diagnosis of intracranial or spinal, localised or metastatic ependymoma according to local pathologist (all WHO grades) including: myxopapillary ependymoma, ependymoma (papillary, clear-cell, tanycytic), ependymoma RELA fusion-positive or anaplastic ependymoma, - Delivery to national referral pathology center of formalin-fixed/paraffin embedded (FFPE) tumour tissue blocks (or at least twenty 5 µm sections on charged slides with sufficient interpretable material and at least ten 10 µm curls in an Eppendorf tube), - Written informed consent (specific to staging) for data and biological samples collection, - All patients and/or their parents or legal guardians willing and able to comply with protocol schedule and agree to sign a written informed consent, - Patients must be affiliated to a health insurance in countries where this is mandatory.   **Exclusion criteria**   - Patient with subependymomas and ependymoblastomas, - Primary diagnosis predating the activation of the SIOP Ependymoma II (SIOP EPII) program (Apr 29^th^ 2015). | | | |
| **Stratum I** | **Stratum II** | **Stratum III** | **Observational stratum** |
| **Inclusion criteria**  • Age >12 months and <22 years at time of study entry,  • No residual measurable ependymoma based on the central neuro-radiological review. This includes:  - R0: No residual tumour on postoperative MRI in accordance with the neurosurgical report  - R1: No residual tumour on MRI but description of a small residual tumour by the neurosurgeon.  - R2: small residual tumour on MRI with the maximum diameter below 5mm in any direction  • Newly diagnosed intracranial ependymoma of WHO grade II-III confirmed by central pathological review,  • No metastasis on spinal MRI and on CSF cytology assessments (see section Mandatory Lumbar puncture),  • No previous radiotherapy,  • No previous chemotherapy (except steroids),  • No co-existent unrelated disease (e.g. renal, haematological) at the time of study entry that would render the patient unable to receive chemotherapy,  • No medical contraindication to radiotherapy, and chemotherapy,  • No signs of infection,  • Adequate bone marrow function:  - WBC >2 x109/l  - Peripheral absolute neutrophil count (ANC) ≥1 x 109/l  - Platelet count ≥100 x 109/l, Haemoglobin >8.0 g/dl (may have PRBC transfusion to achieve Hb >8g/dl)  •Adequate liver function:  - Aspartate aminotransferase (AST) and/or alanine aminotransferase (ALT) 3x institutional ULN for age  - Total bilirubin 2x institutional ULN for age  •Adequate renal function: <1.5x Normal serum creatinine as adjusted for age:  Age Maximum serum creatinine (mg/dl)   - <5 years:0.8 - 5<age<10 years: 1.0 - 10<age<15 years: 1.2 - >15 years: 1.5   Age Maximum serum creatinine (µmol/l)   - <5 years: 46 - 5<age<10 years: 51 - 10<age<15 years: 60 male, 55 female - >15 years: 80 male, 70 female   In case of suspected compromised renal function, glomerular filtration should be measured by an isotope GFR method such as EDTA clearance or by creatinine clearance. GFR should be:   - >80 ml/min/1.73m² for children 2 years and over, - >70 ml/min/1.73 m² for children aged 18-23 months, - >65 ml/min/1.73 m² for children aged 12-17 months,   • Post-menarchal female not pregnant or nursing (breast feeding) and with a negative beta-HCG pregnancy test prior to commencing the trial,  • Males and females of reproductive age and childbearing potential with effective contraception (see Appendix 7 - Definition of highly effective methods of contraception) for the duration of their treatment and 6 months after the completion of their treatment,  • Patients and/or their parents or legal guardians must be willing and able to comply with scheduled visits, treatment plan, laboratory tests and other study procedures of the stratum I,  • Written informed consent (specific to stratum I) from patients and/or their parents or legal guardian(s) obtained for study participation, data acquisition and transfer, collection and transfer of biological samples. Assent, of the child, when appropriate, will be obtained according to institutional guidelines,  • Patients must be affiliated to a health insurance in countries where this is mandatory. | **Inclusion criteria**  • Age >12 months and <22 years at time of study entry,  • Residual non reoperable measurable ependymoma based on central neuro-radiological review (R3-4 residual tumour that can be measured in three planes, for definition of residuum please refer to radiological assessment: (Table 1):  -R3: Residual tumour that can be measured in 3 planes,  -R4: Size of the residual tumour not differing from the preoperative status (e.g. after biopsy)  • Newly diagnosed intracranial ependymoma of WHO grade II-III confirmed by central pathological review,  • No metastasis on spinal MRI and on CSF cytology assessments (see section Lumbar puncture),  • No previous radiotherapy,  • No previous chemotherapy (except steroids),  • No co-existent unrelated disease (e.g. renal, haematological) at the time of study entry that would render the patient unable to receive chemotherapy,  • No medical contraindication to radiotherapy, and chemotherapy,  • No signs of infection,  • Adequate bone marrow function:  - WBC >2 x109/l  - Peripheral absolute neutrophil count (ANC) ≥1 x 109/l  - Platelet count ≥ 100 x 109/l, Haemoglobin > 8.0 g/dl (may have PRBC transfusion to achieve Hb > 8g/dl)  • Adequate liver function:   - Aspartate aminotransferase (AST) and/or alanine aminotransferase (ALT) 3x institutional ULN for age - Total bilirubin 2x institutional ULN for age   • Adequate renal function: <1.5x Normal serum creatinine as adjusted for age:  Age Maximum serum creatinine (mg/dl)   - <5 years 0.8 - >5 <10 years 1.0 - >10 <15 years 1.2 - >15 years 1.5   Age Maximum serum creatinine (µmol/l)   - <5 years: 46 - 5<age<10 years: 51 - 10<age<15 years: 60 male, 55 female - >15 years: 80 male, 70 female   In case of suspected compromised renal function, glomerular filtration should be measured by an isotope GFR method such as EDTA clearance or by creatinine clearance. GFR should be:   - >80 ml/min/1.73m² for children 2 years and over, - >70 ml/min/1.73 m² for children aged 18-23 months, - >65 ml/min/1.73 m² for children aged 12-17 months,   • Post-menarchal female not pregnant or nursing (breast feeding) and with a negative beta-HCG pregnancy test prior to commencing the trial,  • Males and females of reproductive age and childbearing potential with effective contraception (see Appendix 7 - Definition of highly effective methods of contraception) for the duration of their treatment and 6 months after the completion of their treatment,  • Patients and/or their parents or legal guardians must be willing and able to comply with scheduled visits, treatment plan, laboratory tests and other study procedures of the stratum 2,  • Written informed consent (specific to stratum II) from patients and/or their parents or legal guardian(s) obtained for study participation, data acquisition and transfer, collection and transfer of biological samples. Assent, of the child, when appropriate, will be obtained according to institutional guidelines,  •Patients must be affiliated to a health insurance in countries where this is mandatory. | **Inclusion criteria**  • Children younger than 12 months at time of entry to study or any patient ineligible to receive radiotherapy due to age at diagnosis, tumour location or clinician / parent decision and according to national criteria,  • Newly diagnosed intracranial ependymoma of WHO grade II-III confirmed by central pathological review,  • Adequate bone marrow function:  - WBC >2 x109/l  -Peripheral absolute neutrophil count (ANC) ≥1x 109/l  - Platelet count ≥100 x 109/l, Haemoglobin >8.0 g/dl (may have PRBC transfusion to achieve Hb >8g/dl)  • Adequate liver function:  -Aspartate amino-transferase (AST) and/or alanine aminotransferase (ALT) 3x institutional ULN for age  -Total bilirubin 2x institutional ULN for age  • Adequate renal function: <1.5x Normal serum creatinine as adjusted for age:  Age Maximum serum creatinine (mg/dl)  <5 years: 0.8  Age Maximum serum creatinine (µmol/l)  <5 years: 46  In case of suspected compromised renal function, glomerular filtration should be measured by an isotope GFR method such as EDTA clearance or by creatinine clearance. GFR should be:   - >80 ml/min/1.73m² for entry for children 2 years and over, - >70 ml/min/1.73 m² for children aged 18-23 months, - >65 ml/min/1.73 m² for children aged 12-17 months, - >55 ml/min/1.73 m² for children of 6-11 months of age inclusive, - >40 ml/min/1.73 m² for children of 0-5 months of age inclusive.   • No previous chemotherapy (except steroids),  • No previous radiotherapy,  • No co-existent unrelated disease (e.g. renal, haematological) at the time of study entry that would render the patient unable to receive chemotherapy,  • No medical contraindication to chemotherapy,  • No signs of infection,  • Patients and/or their parents or legal guardians must be willing and able to comply with scheduled visits, treatment plan, laboratory tests and other study procedures of the stratum III,  • Written informed consent (specific to stratum III) from parents or legal guardian(s) obtained for study participation, data acquisition and transfer, collection and transfer of biological samples. Assent, of the child, when appropriate, will be obtained according to institutional guidelines,  • Patients must be affiliated to a health insurance in countries where this is mandatory. | - Patients who are not eligible to any of the 3 interventional studies or who refuse to comply with protocol specifications of the interventional studies proposed,   *Nota Bene*: Patients for whom imaging remains RX despite all effort to clarify the MRI conclusion (Table 1) and could not be recruited in stratum III can be included in the observational study   - Main residence in one of the participating countries, - Age below 22 years old at the diagnosis, - Histological diagnosis of intracranial or spinal, localised or metastatic, ependymoma according to local pathologist (all WHO grades) including: myxopapillary ependymoma, ependymoma (papillary, clear-cell, tanycytic), ependymoma RELA fusion-positive and anaplastic ependymoma, confirmed by central pathological review   *Nota Bene: Any* previously unregistered relapsing patients with primary diagnosis posterior to the study activation (Apr 29^th^ 2015) will be eligible.   - All patients and/or their parents or legal guardians must receive an information sheet. Assent of the child, when appropriate, as well as written consent of parents or legal guardians will be obtained according to institutional / national guidelines. |
| **Exclusion criteria**   - Tumour entity other than primary intracranial ependymoma, - Patients with WHO grade I ependymoma including myxopapillary variant, - Patients with spinal cord location of the primary tumour, - Participation in another trial for treatment of ependymoma, - Concurrent treatment with any anti-tumour agents, - Inability to tolerate chemotherapy, - Unable to tolerate intravenous hydration, - Other severe acute or chronic medical or psychiatric conditions or laboratory abnormalities that may increase the risk associated with study participation or investigational product administration, or may interfere with the interpretation of study results in the judgment of the investigator, - Pre-existing mucositis, peptic ulcer, inflammatory bowel disease, ascites, or pleural effusion,   Contraindication to one of the Investigational Medicinal Product (IMP) according to Summary of product characteristics (SmPCs), Medicine and Healthcare products Regulatory agency.   - Patient for whom imaging remains RX despite all effort to clarify the MRI conclusion (Table 1): | **Exclusion criteria**  • Tumour entity other than primary intracranial ependymoma,  • Patients with WHO grade I ependymoma including myxopapillary variant,  • Patients with spinal cord location of the primary tumour,  • Participation in another trial for treatment of ependymoma,  • Concurrent treatment with any anti-tumour agents,  • Inability to tolerate chemotherapy,  • Unable to tolerate intravenous hydration,  • Other severe acute or chronic medical or psychiatric conditions or laboratory abnormalities that may increase the risk associated with study participation or investigational product administration, or may interfere with the interpretation of study results in the judgment of the investigator,  • Pre-existing mucositis, peptic ulcer, inflammatory bowel disease, ascites, or pleural effusion,  • Contraindication to one of the IMP according to the SmPCs Medicine and Healthcare products Regulatory agency.  • Patient for whom imaging remains RX despite all effort to clarify the MRI conclusion (Table 1): | **Exclusion criteria**  • Tumour entity other than primary intracranial ependymoma,  • Patients with WHO grade I ependymoma including myxopapillary variant,  • Patients with spinal cord location of the primary tumour,  • Participation in another trial for treatment of ependymoma,  • Concurrent treatment with any anti-tumour agents,  • Inability to tolerate chemotherapy,  • Unable to tolerate intravenous hydration,  • Other severe acute or chronic medical or psychiatric conditions or laboratory abnormalities that may increase the risk associated with study participation or investigational product administration, or may interfere with the interpretation of study results in the judgment of the investigator,  • Pre-existing mucositis, peptic ulcer, inflammatory bowel disease, ascites, or pleural effusion,  • Pre-existing severe hepatic and/or renal damage,  • Family history of severe epilepsy in immediate family siblings,  • Presence of previously undiagnosed mitochondrial disorder detected by screening as part of trial,  • Elevated blood ammonium level ≥1.5xULN,  • Elevated blood lactate level ≥1.5xULN,  • Contraindication to one of the IMP used according to the SmPCs Medicine and Healthcare products Regulatory agency. | **Exclusion criteria**  • Tumour entity other than primary ependymoma  • Patient with subependymomas and ependymoblastomas.  • Primary diagnosis predating the activation of the SIOP EPII program (Apr 29th 2015) |

*SmPCs as defined in the UK were chosen for the assessment of safety

**Supplementary Table S3. Detailed chemotherapy in 1) Stratum I; 2) Stratum II; 3) Stratum III.**

**1) STRATUM I. 1A). Detailed chemotherapy**

| **STRATUM I - VEC+ cisplatin course** | |
| --- | --- |
| **Week 1**  **Days 1-3** | **D1: Vincristine (VCR)** 1.5 mg/m² (maximal dose 2 mg) as an i.v. bolus  **D1-D3: Etoposide (VP16)** 100 mg/m² infused over 1–4 hours according to standard institutional practice.  **D1: Cyclophosphamide** 3000 mg/m² in 3 divided infusions (1000 mg/m²/infusion) infused over 60 minutes at eight hourly intervals. |
| **Week 4**  **Day 22** | **CISPLATIN (CDDP)** 80 mg/m² as an i.v. infusion over 4 hours.  **VINCRISTINE (VCR)** 1.5 mg/m² (maximal dose 2 mg) i.v. bolus |
| **Week 6**  **Days 36-38** | **D36:** **Vincristine (VCR)** 1.5 mg/m² (maximal dose 2 mg) as an i.v. bolus  **D36-D38**: **Etoposide (VP16)** 100 mg/m² infused over 1–4 hours according to standard institutional practice.  **D36:** **Cyclophosphamide** 3000 mg/m² in 3 divided infusions (1000 mg/m²/infusion) infused over 60 minutes at eight hourly intervals. |
| **Week 9**  **Day 57** | **CISPLATIN (CDDP)** 80 mg/ m² as an i.v. infusion over 4 hours...  **VINCRISTINE (VCR)** 1.5 mg/m² (maximal dose 2 mg) i.v. bolus |
| **Week 11**  **Days 71-73** | **D71: Vincristine (VCR)** 1.5 mg/m² (maximal dose 2 mg) as an i.v. bolus  **D71-D73 Etoposide (VP16)** 100 mg/m² infused over 1–4 hours according to standard institutional practice.  **D71 Cyclophosphamide:** 3000 mg/m² in 3 infusions (1000 mg/m²/infusion) infused over 60 minutes at eight hourly intervals. |
| **Week 14**  **Day 92** | **CISPLATIN (CDDP)** 80 mg/ m² as an i.v. infusion over 4 hourss  **VINCRISTINE (VCR)** 1.5 mg/ m² (maximal dose 2 mg) i.v. bolus |
| **Week 16**  **Days 106-108** | **D106: Vincristine (VCR)** 1.5 mg/m² (maximal dose 2 mg) as an i.v. bolus  **D106-D108 Etoposide (VP16**) 100 mg/m² infused over 1–4 hours according to standard institutional practice.  **D106 Cyclophosphamide** 3000 mg/m² in 3 infusions (1000 mg/m²/infusion) infused over 60 minutes at eight hourly intervals. |

**2) STRATUM II. Post-surgical induction chemotherapy A) VEC+HD-MTX, B) VEC schedule, and C) Maintenance therapy**

**2A)**

| **STRATUM II - VEC+HD-MTX course** | |
| --- | --- |
| **Week 1**  **Days 1-3** | **D1: Vincristine (VCR)** 1.5 mg/m² (maximal dose 2 mg) as an i.v. bolus  **D1-D3 Etoposide (VP16)** 100 mg/m² infused over 1–4 hours according to standard institutional practice.  **D1 Cyclophosphamide** 3000 mg/m² in 3 divided infusions (1000 mg/m²/infusion) infused over 60 minutes at eight hourly intervals. |
| **Week 3**  **Day 15** | **Methotrexate** 8000mg/m² as an i.v. infusion over 24 hours  10% of the dose should be given over the first hour and the remaining 90% over the following 23 hours. The second infusion must finish at T=25 (24 hours after the first infusion was started) even if it has not been completed at that time. |
| **Week 4**  **Days 22-24** | **D22: Vincristine (VCR)** 1.5 mg/m² (maximal dose 2 mg) as an i.v. bolus  **D22-D24: Etoposide (VP16)** 100 mg/m² infused over 1–4 hours according to standard institutional practice.  **D22: Cyclophosphamide** 3000 mg/m² in 3 divided infusions (1000 mg/m²/infusion) infused over 60 minutes at eight hourly intervals. |
| **Week 6**  **Day 36** | **Methotrexate** 8000mg/m² as an i.v. infusion over 24 hours.  10% of the dose should be given over the first hour and the remaining 90% over the following 23 hours. The second infusion must finish at T=25 (24 hours after initiation of the first infusion) even if it has not been completed at that time. |
| **Week 7**  **Days 43-45** | **D43: Vincristine (VCR)** 1.5 mg/m² (maximal dose 2 mg) as an i.v. bolus  **D43-D45: Etoposide (VP16)** 100 mg/m² infused over 1–4 hours according to standard institutional practice.  **D43: Cyclophosphamide** 3000 mg/m² in 3 divided infusions (1000 mg/m²/infusion) infused over 60 minutes at eight hourly intervals. |
| **Week 9**  **Day 57** | **Methotrexate** 8000mg/m² as an i.v. infusion over 24 hours.  10% of the dose should be given over the first hour and the remaining 90% over the following 23 hours. The second infusion must finish at T=25 (24 hours after the first infusion was started) even if it has not been completed at that time. |

**2B)**

| **VEC course** | |
| --- | --- |
| **Week 1**  **Days 1-3** | **D1: Vincristine (VCR)** 1.5mg/m² (Maximum dose: 2mg) as an i.v. bolus  **D1-D3: Etoposide (VP16)** 100mg/m² infused over 1 – 4 hours according to standard institutional practice.  **D1: Cyclophosphamide** 3000mg/m² in 3 divided infusions (1000 mg/m²/infusion) infused over 60 minutes at eight hourly intervals. |
| **Week 4**  **Days 22-24** | **D22: Vincristine (VCR)** 1.5mg/m² (Maximum dose: 2mg) as an i.v. bolus  **D22-D24: Etoposide (VP16)** 100mg/m² infused over 1–4 hours according to standard institutional practice.  **D22: Cyclophosphamide** 3000mg/m² in 3 divided infusions (1000 mg/m²/infusion) infused over 60 minutes at eight hourly intervals. |
| **Week 7**  **Days 43-45** | **D43: Vincristine (VCR)** 1.5mg/m² (Maximum dose: 2mg) as an i.v. bolus  **D43-D45: Etoposide (VP16)** 100mg/m² infused over 1–4 hours according to standard institutional practice.  **D43: Cyclophosphamide** 3000mg/m² in 3 divided infusions (1000 mg/m²/infusion) infused over 60 minutes at eight hourly intervals. |

### 2C) Maintenance chemotherapy

| **VEC+ cisplatin course** | |
| --- | --- |
| **Week 1**  **Days 1-3** | **D1: Vincristine (VCR)** 1.5 mg/m² (maximal dose 2 mg) as an i.v. bolus  **D1-D3: Etoposide (VP16)** 100 mg/m² infused over 1–4 hours according to standard institutional practice.  **D1: Cyclophosphamide** 3000 mg/m² in 3 divided infusions (1000 mg/m²/infusion) infused over 60 minutes at eight hourly intervals. |
| **Week 4**  **Day 22** | **CISPLATIN (CDDP)** 80 mg/m² as an i.v. infusion over 4 hours.  **VINCRISTINE (VCR)** 1.5 mg/m² (maximal dose 2 mg) i.v. bolus |
| **Week 6**  **Days 36-38** | **D36:** **Vincristine (VCR)** 1.5 mg/m² (maximal dose 2 mg) as an i.v. bolus  **D36-D38**: **Etoposide (VP16)** 100 mg/m² infused over 1–4 hours according to standard institutional practice.  **D36:** **Cyclophosphamide** 3000 mg/m² in 3 divided infusions (1000 mg/m²/infusion) infused over 60 minutes at eight hourly intervals. |
| **Week 9**  **Day 57** | **CISPLATIN (CDDP)** 80 mg/m² as an i.v. infusion over 4 hours...  **VINCRISTINE (VCR)** 1.5 mg/m² (maximal dose 2 mg) i.v. bolus |
| **Week 11**  **Days 71-73** | **D71: Vincristine (VCR)** 1.5 mg/m² (maximal dose 2 mg) as an i.v. bolus  **D71-D73 Etoposide (VP16)** 100 mg/m² infused over 1–4 hours according to standard institutional practice.  **D71 Cyclophosphamide** 3000 mg/m² in 3 divided infusions (1000 mg/m²/infusion) infused over 60 minutes at eight hourly intervals. |
| **Week 14**  **Day 92** | **CISPLATIN (CDDP)** 80 mg/ m² as an i.v. infusion over 4 hours…  **VINCRISTINE (VCR)** 1.5 mg/ m² (maximal dose 2 mg) i.v. bolus |
| **Week 16**  **Days 106-108** | **D106: Vincristine (VCR)** 1.5 mg/m² (maximal dose 2 mg) as an i.v. bolus  **D106-D108 Etoposide (VP16**) 100 mg/m² infused over 1–4 hours according to standard institutional practice.  **D106 Cyclophosphamide** 3000 mg/m² in 3 divided infusions (1000 mg/m²/infusion) infused over 60 minutes at eight hourly intervals. |

**3) STRATUM III. 3A) Post-operative intensive chemotherapy, 3B) Dosing schedule schedule.** HDACi: histone deacetylase inhibitor (Valproate); MTX: methotrexate

**3A)**

| **Chemotherapy +/- HDACi = valproate (**)** | | | | | | | |
| --- | --- | --- | --- | --- | --- | --- | --- |
| **CYCLE N** | **1** | **2** | **3** | **4** | **5** | **6** | **7** |
| Vincristine-Carboplatin | D1 | D57 | D113 | D169 | D225 | D281 | D337 |
| Vincristine-MTX | D15 | D71 | D127 | D183 | D239 | D295 | D351 |
| Vincristine-Cyclophosphamide | D29 | D85 | D141 | D197 | D253 | D39 | D365 |
| Cisplatin 2-day  Continuous infusion | D43-44 | D99-100 | D154-155 | D211-212 | D267-268 | D323-324 | D379-380 |
| +/- **HDACi**= valproate (*) | Initial dose: 30 mg/kg/day for two weeks divided in 2 doses (15mg/kg/b.d.). Increasing weekly up to 40->50->60 mg/kg/day divided in 2 doses until serum target level achieved. If therapeutic levels of 100-150 µg/ml are not achieved with 60 mg/kg/day administered in three divided doses (20mg/kg/dose b.d.). If therapeutic level is still not achieved, increase to higher total daily dose in 5 mg/kg/day increments i.e. 65 mg/kg/day then 70 mg/kg/day (23.3 mg/kg/dose t.d.s) divided in 3 doses. | | | | | | |

**3B)**

| **Dosing schedule (***)** | **Dose for infants over 12 months** | **Dose for infants**  **6 to 12 months** | **Dose for infants**  **less than 6 months** |
| --- | --- | --- | --- |
| **Vincristine**  **(Maximum dose: 2mg)** | 1.5 mg/m² x 1 | 1.125 mg/m² x 1 | 0.75 mg/m² x 1 |
| **Carboplatin** | 550 mg/m²x 1 | 412.5 mg/m² x 1 | 275 mg/m² x 1 |
| **MTX** | 8000 mg/m² x 1 | 6000 mg/m² x 1 | 4000 mg/m² x 1 |
| **Cyclophosphamide** | 1500 mg/m² x 1 | 1125mg/m² x 1 | 750 mg/m² x 1 |
| **Cisplatin** | 40 mg/m² x 2 | 30 mg/m² x 2 | 20 mg/m² x 2 |
| +/- **HDACi=** valproate **(*)** | 30 mg/kg/day | 30 mg/kg/day | 30 mg/kg/day |

* Initial dosing then according to monitoring

** If residual disease please consider for further surgery at each reassessment point.

*** For patients aged:

- 12 months and over: full surface-area-based dose of chemotherapy
- 6-11 months: 75% of the surface-area-based dose of chemotherapy
- Under 6 months: 50% of the surface-area –based dose of chemotherapy

**Supplementary Table S4: Participating countries, national coordinators, sites, and activation dates in each country and number of centers.** Greece, Poland, Slovenia, and Sweden are planned to be activated.

| **Country** | **Investigator/**  **National coordinator** | **Site** | **Activation date** | **Activated centers** |
| --- | --- | --- | --- | --- |
| France | Dr Pierre Leblond | Institut d’Hématologie et d’Oncologie Pédiatrique, Lyon | April 2015 | 27/27 |
| United Kingdom | Dr Richard Grundy | Children's Brain Tumour Research Centre D 32, Medical School, Queen's Medical Centre, Nottingham | December 2015 | 20/20 |
| Italy | Dr Maura Massimino | Fondazione IRCCS Istituto Nazionale dei Tumori, Milan | January 2016 | 14/14 |
| Belgium | Dr Caroline Piette | Département Hémato-oncologie pédiatrique, CHR de la Citadelle, Liège | February 2016 | 8/8 |
| Czech Republic | Dr Jaroslav Sterba | Paediatric Oncology Department, University Hospital Brno | May 2017 | 2/4 |
| Spain | Dr. Ana Fernández-Teijeiro | Paediatríc Oncology Program, Hospitales Universitarios Virgen Macarena y Virgen del Rocío, Sevilla | October 2017 | 18/20 |
| Ireland | Dr Michael Capra | Our Lady’s Children’s Hospital  Haematology and Oncology department, Dublin | November 2017 | 1/9 |
| Austria | Dr Martin Benesch | Division of Paediatric Haematology/Oncology, Department of Paediatrics and Adolescent Medicine, Medical University of Graz, Graz | May 2018 | 6/6 |
| Switzerland | Dr Nicolas Gerber | Department of Oncology, University Children’s Hospital, Zurich | May 2018 | 9/9 |
| Finland | Dr Kirsti Sirkiä | Division of Haematology - Oncology and Stem Cell Transplantation Children's hospital, Helsinki University Central Hospital, Hus | May 2018 | 1/9 |
| Germany | Dr Stefan Rutkowski | Department of Paediatric Haematology and Oncology, University Medical Center Hamburg-Eppendorf, Hamburg | August 2018 | 48/57 |
| The Netherlands | Dr Jasper van der Lugt | Princess Maxima Center for paediatric oncology, Utrecht | March 2020 | 1/2 |
| Denmark | Dr Ines Ackerl Kristensen | Paediatric oncology, Aarhus University Hospital, Aarhus | November 2020 | 2/3 |
| Norway | Dr Ingrid Kristin Torsvik | Department of Paediatrics, Haukeland University Hospital, Bergen | December 2020 | 3/4 |
| Greece | Dr Vita Ridola | Department of Paediatric Haematology and Oncology, Children's Hospital MITERA, Athens |  |  |
| Poland | Dr Marta Perek-Polnik | Klinika Onkologii IP-CZD, The Children's Memorial Health Institute CMHI (IP-CZD), Warsaw |  |  |
| Slovenia | Dr Lidija Kitanovski | Department of Paediatrics-Division of Haemato-oncology, University Medical Center Ljubljana, Ljubljana |  |  |
| Sweden | Dr Helena Mörse | Paediatric Oncology Department, Skane University Hospital, Lund |  |  |

**Supplementary Figure S5. Trial status as of August 31, 2021.** cRT: conformal focal radiotherapy; CT: Chemotherapy; VEC: combination of vincristine-etoposide-cyclophosphamide; HD-MTX: High-dose methotrexate; HDACi: histone deacetylase inhibitor (Valproate).

**Observational study**

without staging

**N= 21**

**Observational study**

after staging

**N= 187**

**STAGING**

**N= 559**

**(ongoing N= 20)**

**Patients with newly diagnosed ependymoma**

**N= 580**

**Patients excluded**

**N= 46**

- 19 Other diagnosis (after central review)
- 6 consent withdrawal
- 8 family decision
- 11 investigator’s decision
- 1 progression during staging
- 1 spinal metastases

**STRATUM III**

**N= 47** /100 patients expected

**Standard CT**

**N= 23**

**Standard CT**

**+ HDACi**

**N= 24**

**STRATUM I**

**N= 224** /320 patients expected

**cRT**

**+ Observation**

**N= 112**

**cRT**

**+ Maintenance CT**

**N= 112**

**VEC**

**N= 17**

**VEC**

**+ HD-MTX**

**N= 18**

**STRATUM II**

**N= 35** /60 patients expected
